# Molecular docking analysis of GC-MS analyzed bioactive compounds from the rhizome of Hedychium rubrum with four protein targets

**DOI:** 10.6026/97320630018943

**Published:** 2022-10-31

**Authors:** Ksh Sangeeta Devi, M Damayanti Devi, N Chetan Das, D Velmurugan, N Rajen Singh

**Affiliations:** 1 Manipur University, Manipur, India; 2 AMET University, Kanathur, ECR road, Chennai, Tamilnadu, India

**Keywords:** antimicrobial, plant extract, software and phytoconstituents

## Abstract

Hedychium rubrum, a traditional medicinal plant of Manipur belonging to the family Zingeberaceae was screened for its biological activity. The methanolic extract of its rhizome was prepared by Soxhlet extraction method and was further subjected to GC-MS to know its bioactive compounds. The in vitro antimicrobial activity assay was tested against five bacteria causing UTI. Klebseilla pneumoniae showed most sensitive followed by Pseudomonas aeruginosa, Escherichia coli, Staphylococcus aureus and Enterococcus faecalis in the order. Plant extract showed higher inhibition zone than the positive control used. According to the higher quality of compounds from the GCMS results nine compounds were selected for further in silico studies using GOLD software against four protein targets. The phytoconstituents present in the methanolic extract have the ability to bind at the receptor site of all four targeted proteins. ADMET and TOPKAT studies were also carried out.

## Background:

Plants play a vital role in the treatment and prevention of many diseases and also reduce the risk of adverse effect compared to the conventional treatment [1]. From ancient times plants are being used as medicine for their usefulness in human history [2]. The identification of biologically active compounds present in the plant which will lead to the pharmacological studies is the most essential part in the scientific investigation [3-5]. Hedychium J.Koeng, commonly known as "ginger lily or butterfly lily" is one of the beautiful and ornamental flowers, blooming of the Zingeberaceae family [6]. Flowers have different colours and are short lived. The medicinal properties and the horticulture significance lead to its wide cultivation [7]. The genus comprises approximately 80 species throughout distributed in tropical Asia to NewGuinea, Australia, the Solomon Islands, New Hebrides, New Caledonia, Fiji and Samoa [8-9]. With about 44 taxa [10], Hedychium is the largest genus found in India belonging to the Zingeberaceae family and its 16 endemics [11] are widespread in most of the Northeastern states of India. Hedychium is believed to be originated from Northeast India [12] (24 spp) and is found with 65 valid taxons in the world [13]. Hedychium, a perennial ornamental plant is highly valued of its showy flowers myriad hues and fragrances. Essential oil is extracted from its flowers and rhizomes [14]. Stems contain 43-48% cellulose which is useful in making paper [15]. Manipur, a Northeastern state of India situated between latitude 23.80N-25.68N and longitude 93.03E-94.78E coves a total geographical area of 22,327.sq.km and constitutes only 0.7% of the total land surface of India. 9/10 of the total area constitutes hills which surrounds the remaining 1/10 valley area. In Manipur, Hedychium is one of the favourable parts of local cuisines rather than its edibility. It has a sacred ritual component and ethno botanical importance. Though it has highly significant uses, still it is a grossly underutilized crop [16]. Hedychium rubrum, one of the species which is known as "red ginger lily" (Manipuri local name: Takhelei angangba) is selected in this study. This species had been used by traditional healers in Manipur. Computer aided drug design (CADD) is an efficient method of drug discovery, including virtual screening and pharmacophore design [17]. It can overcome certain difficulties from experiments in the laboratory and CADD can give a virtual approach with a relatively low cost [18]. We have conducted research using the rhizome of Hedyhcium rubrum as low-cost, renewable bioactive natural product sources. Therefore, it s of interest to carry out the screening of phytochemical components, in vitro antibacterial assay against UTI causing bacteria and docking study with ADMET and Topkat properties against the four targeted proteins.

## Materials and Methods:

### Collection of plant materials:

The study sample has been collected from different parts i.e., Imphal East, Imphal West and Bishnupur districts of the Manipur. Rhizomes of the plant part were used.

### Preparation of plant:

The rhizomes of Hedychium rubrum were used in this study. Rhizome more than 1 year old was collected. The samples were washed with tap water and then with distilled water. They were then cut into slices and left for shade air dry for 20 days in an airy room. The air dried samples were used in further studies.

### Preparation of plant extract:

To prepare the methanolic extract, 150gm of the dried sample was subjected to extraction with the 1.5 L solvent at 60°C using Soxhlet extractor. The extraction process was continued for 24 hours. The crude extract was separated using Rotor vapour evaporator. Crude extract was collected and kept in refrigerator at 4°C for further used.

### GC-MS analysis:

GC MS (Agilent, USA), coupled with a (7890B- GC and 5977A MSD) Agilent mass selective detector (Triple-Axis Detector) was used for detecting the compounds. The GC-MS system was equipped with a HP-5MS 5% Phenyl methyl silox column (30 m x 250 µm x 0.25 µm film thickness). Analyses were carried out using helium as carrier gas at a flow rate of 1 ml/min and a split ratio of 1:1 using the following conditions, 95 min total and run time temperature program: program starts with 50°C for 2 min hold, then ramped 3°C/min up to 270°C, then hold for 20 min at same temperature. 1µl of diluted sample was injected to column and mass spectra were obtained by electron impact ionization 70eV at scan range 40-700 m/z. Compounds were identified by matching of their mass spectra (NIST library).

### Preliminary phytochemical tests:

#### Test for tannins: 

About 0.5g of the dried powdered sample was boiled in 20ml of water in a test tube and then filtered. Few drops of 0.1% ferric chloride were added and observed for blue-black colouration [19].

#### Test for phlobatannins:

Deposition of a red precipitate is seen when an aqueous extract of the plant sample was boiled with 1% aqueous hydrochloric acid. The presence of red precipitate was taken as an evidence for the presence of phlobatannins [20].

#### Test for saponnins:

About 2g of the powdered sample was boiled in 20ml of distilled water in a water bath and filtered. 10 ml of the filtrate was mixed with 5ml of distilled water and shaken vigorously until persistent froth. The froth was mixed with 3 drops of olive oil and shaken vigorously, then observed for the formation of emulsion [21].

#### Test for flavonoids: 

5ml of dilute ammonia solution was added to a portion of the aqueous filtrate of plant extract followed by addition of concentrated H2SO4. A yellow colouration observed in each extract indicated the presence of flavonoids. The yellow colouration disappeared on standing [22].

#### Test for terpenoids:

5 ml of each extract was mixed in 2ml of chloroform, and concentrated H2SO4 (3ml) was added carefully to form a layer. A reddish brown colouration of the inter face was formed to show the presence of terpenoids [23].

#### Test for cardiac glycosides:

5 ml of each extract was treated with 2ml of glacial acetic acid containing one drop of ferric chloride solution was added followed by H2SO4 along the side of the test tube. The formation of brown ring at the interface gives positive indication for cardiac glycosides and violet ring appeared below the brown ring [24].

#### Test for phenols:

To 1ml of extract sample, 2ml of distilled water followed by few drops of 10% aqueous ferric chloride solution were added. Formation of bluish black colour indicated the presence of phenols [25].

### In vitro antimicrobial activity assay:

Antibacterial activity was determined by standard filter-disc diffusion technique. The in vitro antimicrobial activity was performed against overnight grown cultures of five selected bacteria, namely, gram negative bacteria (Klebsiella pneumoniae, Escherichia. coli, and Pseudomonas aeruginosa) and gram positive bacteria (Staphylococcus aureus, and Enterococcus. faecalis). Gentamicin was used as positive control in this experiment. The bacterial cultures were maintained on slants consisting of nutrient agar medium. 48 hour cultures of 5 organisms were used in the in vitro antimicrobial activity. In this as say, 0.5 mg of each extract was dissolved separately in 1ml sterile of dimethyl sulfoxide (DMSO).Nutrient agar medium was prepared and sterilized by autoclave. In an aseptic room, inside the laminar flow they were poured onto sterile petridishes to a uniform depth of 3mm and then allowed to solidify at room temperature for overnight for checking of contamination. Next day after solidification, the test organisms were inoculated with the help of L-shape spreader (the suspensions culture of bacteria).This provides the uniform surface growth of bacterium and is used for antibacterial sensitivity studies. The wells (6mm in diameter) were dug in the media with the help of a sterile metallic borer. The recommended test sample (0.5gm/1ml in DMSO) was introduced in the respective wells. The plates were incubated immediately at 37°C for 48 hrs. Microbial growth inhibition was determined by measuring the diameter of the zone of inhibition which was assessed at 48hrs incubation.

### Molecular Docking Studies:

Receptor and its binding site: The three dimensional structures of four receptors such as EGFR (PDB ID: 1M17), DNA gyrase (PDB ID: 5L3J), Uromucoid (PDB ID: 5FBH) and Caspase 3V266F (PDB ID: 5IAE) were retrieved from PDB database [26]. To determine the binding affinities between ligand and receptor, the amino acids in the binding pockets were predicted by Q-site finder [27].

### Ligand modelling:

The structures (SD) of the identified compounds were sketched using ChemDraw and used as ligands [28]. The SD files were converted to their corresponding three-dimensional (3D) structures and saved as pdb format using Open Babel [29].

## Results and discussion:

### GCMS results:

The GC chromatograms of the three extracts presented in Figure 3(see PDF) shows the retention time in the column and the detected peaks which correspond to the bioactive compounds present in the extract.

### Preliminary phytochemical tests:

The Phytochemical composition of Hedychium rubrum determined is summarised in Table 2(see PDF). The methanolic extract of rhizome shows the presence of saponin, tannins, flavanoids, terpenoids, cardiac glycosides phlabotannines and phenol.

### In vitro antimicrobial activity assay:

The above histogram showed the invitro antimicrobial activity of methanol extract of Hedychium rubrum against the five organisms. The plant extract shows significant activity against all the tested organisms. Hedychium rubrum methanol extract showed antimicrobial inhibitory activity against at MIC values of 0.5 mg/mL. Klebseilla pneumoniae (14.4 ± 0.25), showed most sensitive followed by Pseudomonas aeruginosa (12.8 ± 0.37), Escherichia coli (12.4 ± 0.25) Staphylococcus aureus (12 ± 0.45) and Enterococcus faecalis (10.6 ± 0.25) in the order. Gentamicin was used as positive control. The plant extract showed higher inhibition zone than the positive control. However, this study showed the efficacy of plants against human pathogen UTI causing bacteria.

### Molecular docking results:

ADMET:

Nine compounds were accessible according to the results of ADME Solubility Level. The abilities to cross the blood brain barrier (BBB) of HM1, HM2, HM3 were low and HM5, HM6, HM7, HM8, HM9 were undefined while HM4 was highly penetrant. In addition, seven of these compounds were not CYP2D6 inhibitors as per the prediction results, two of these compounds were CYP2D6 inhibitors. Furthermore, these eight compounds were easily absorbed and showed great plasma protein binding ability and one of them was not. Most of the compounds were likely to be highly bound to carrier proteins in the blood. Rat oral maximum lethal dose was also calculated for individual hit compounds and was listed in the Table 4(see PDF). NTP carcinogenicity prediction had been carried out on both female and male rats, and two compounds were found to be carcinogenic. Most of the compounds showed non carcinogenic properties against the female mouse while the others might be carcinogenic in toxicity risk assessment results (Table 5 - see PDF). Furthermore, the Ames mutagenicity and skin irritation tests had been performed against all the nine potential compounds. Among nine of these potential compounds, HM1 and HM3 showed mild mutagenicity. Compounds showed mild and moderate skin irritation test and only one showed severe. Docking study was performed using GOLD to obtain the interactions between the active site of four targeted proteins Table 6b and nine ligands Table 6a. Each ligand was imported on the GOLD software facilitating them to undergo flexible docking commencing all default parameters with each of the four different targets. The best ligand pose was selected according to the affinity towards the amino acids which is denoted by the highest fitness score. The ligand pose was better when the GOLD fitness score is large because it was calculated based on the negative of the sum of the component energy terms. By using optimised fitness function, well fitted ligand binding position that has the least energy with average Gold fitness score was predicted. From Table 6b(see PDF) it is noted that the ligand penta decanoic acid,14- methyl- methyl ester(HM4 sample code) has the highest fitness scores of 60.22 against the EGFR protein target (PDB ID: 1M17). From Figure 5(see PDF) it can be elucidated that the interacting amino acids are LEU149, LEU23, LEU97, ALA48, CYS80, GLU67, and PHE161. Hydrogen bond interaction plays a significant role of the protein structure and biological function, so further analysis was carried out with the ligand pose. It revealed that the PHE161 forms a hydrogen bond with O and GLU67 with O. The same ligand has docked with the uromucoid protein target (PDB ID: 5FBH) and gold fitness score was 33.42. The interacting amino acids at the receptor site are HIS688, VAL692 and PHE673 as shown in Figure 6(see PDF). 1-docosanol methyl ether had the fitness score of 57.56 with the DNA gyrase protein target (PDB ID: 5L3J) with VAL29, VAL 135, VAL57, ALA33, THR133, ILE64, PRO65, ARG62, HIS41 as the interacting amino acids at the receptor site shown in Figure 7(see PDF).In Figure 8(see PDF), Gamma sitosterol (HM8 sample code) has the highest fitness score of 57.54 against the caspase 3 V266F(PDB ID: 5IAE) and the interacting amino acids are ARG410, TRP409, CYS376, TYR407, PHE459.ARG410 forms the hydrogen bond with NH group.

## Conclusions:

Plant derived bioactive molecules which have pharmacogenetic properties are the valuable and viable alternatives. Nowadays, researchers focus their attention in discovering novel plant derived pharmacologically active compounds for treating many diseases primarily. In this study, we report that the methanolic extract of rhizomes of Hedychium rubrum contains phyto compounds which have antibacterial properties and biological properties. The analysis of antimicrobial assay shows the effective zone of inhibition compared to the standard. The results of our present study indicate that the plant extract possesses good antimicrobial activity against 5 UTI causing bacteria. This study also provided the development of ligand-based pharmacophore model by 3D-QSAR Pharmacophore Generation protocol using Discovery Studio. The 9 compounds were subjected to further ADMET studies and were carried out for toxicity assessment studies using TOPKAT program to obtain potent compounds. EGFR and Caspase 3V266F are the protein targets related to the lung cancer and cervical cancer, respectively. Uromucoid is the protein related to the kidney function. DNA gyrase II target is related to the antimicrobial activity of this plant extract. Analysis of the GOLD docking scores and molecular interactions of all the ligands had shown their ability to bind at the active site of the four targeted proteins. The plant used by our traditional healer has thus been shown for its scientific significance towards novel drugs curing the various ailments.
